# Design-Oriented Degradation Mapping and Hyperelastic Model-Switch Guidelines for Nitrile-Butadiene Rubber Seals

**DOI:** 10.3390/polym17172316

**Published:** 2025-08-27

**Authors:** Na-Yeon Choi, Dong-Seok Kim, Sung-Uk Zhang

**Affiliations:** 1 Digital Twin Laboratory, Dong-Eui University, 176 Eomgwang-ro, Busan 47340, Republic of Korea; nyanxun@office.deu.ac.kr; 2 Center for Brain Busan 21, Dong-Eui University, 176 Eomgwang-ro, Busan 47340, Republic of Korea; 3 Korea Atomic Energy Research Institute, Gyeongju-si 38180, Republic of Korea

**Keywords:** NBR, accelerated ageing tests, Arrhenius model, finite element method, hyperelastic model, design guideline

## Abstract

Nitrile-butadiene rubber (NBR) seals used in automotive and energy equipment undergo pronounced mechanical degradation at elevated temperatures, yet a quantitative rule for switching between hyperelastic models remains unclear. Here, accelerated thermal aging tests were linked to service conditions by estimating the activation energy via Flynn–Wall–Ozawa analysis and applying an Arrhenius-based equivalence. Tensile testing, dynamic mechanical analysis, and thermogravimetric analysis were combined to track embrittlement and crosslinking, and finite element simulations were benchmarked against experiments using an L2-norm metric. The outcome is a degradation map with a model-switching guideline. The Neo-Hookean model is preferred in the less-embrittled regime, whereas the five-parameter Mooney–Rivlin model is recommended as embrittlement progresses. This framework improves stress-prediction fidelity while keeping model complexity commensurate with the aging state, enabling faster and more reliable design of NBR seals for high-temperature automotive and renewable-energy applications.

## 1. Introduction

Elastomeric materials, commonly known as rubbers, are indispensable in modern engineering due to their unique properties, such as high elasticity, resilience, and durability [1]. Their applications are vast, ranging from automotive tires and seals, where materials like Styrene-Butadiene Rubber (SBR) and Ethylene Propylene Diene Monomer (EPDM) are common, to advanced aerospace and biomedical devices. Among these versatile materials, Nitrile-Butadiene Rubber (NBR) is widely used as a key sealing material to prevent gas or oil leakage in high-temperature, high-pressure moving parts due to its excellent oil and abrasion resistance [2,3].

However, long-term exposure of NBR to environments with a combination of oxygen, heat, mechanical load, and chemicals accelerates chain scission, further cross-linking, and oxidative degradation, causing a rapid deterioration of its mechanical properties and durability [4,5,6]. As this degradation can lead to the failure of the entire system through pressure loss and lubricant depletion [7], predicting degradation and incorporating it into design is essential.

Given the extremely slow rate of thermal aging in rubber under real-world conditions, significant research has been dedicated to simulating equivalent states of degradation using accelerated aging tests [8,9]. Existing literature can be broadly categorized into three streams: (1) Studies of material degradation mechanisms focusing on changes in chemical structure and crosslink density [10,11,12,13,14,15]; (2) reliability engineering approaches that use accelerated tests to quantify long-term performance and predict service life [16,17,18,19,20,21]; and (3) simulation approaches that seek to enhance the accuracy of Finite Element Analysis (FEA) by fitting material model parameters to experimental data at various stages of degradation [22,23,24,25,26].

Although each research area holds significant merit, a critical gap persists: insights gained at the molecular level are not directly integrated into the selection process for engineering design models. First, molecular or network metrics are characterized but rarely translated into a quantitative rule that selects among constitutive models. Second, accelerated-aging studies establish time–temperature relations yet do not prescribe how specific service conditions should determine model choice. Third, model comparisons are commonly performed at discrete aging states with local fit metrics and limited uncertainty reporting, leaving no clear boundary for a model switch. Consequently, in practical applications, the choice of an optimal model for a given level of degradation often relies on empirical judgment rather than a systematic guideline.

To address this gap, we introduce and experimentally validate an end-to-end workflow that maps service time–temperature inputs to a quantitative rule for model selection in aged NBR. Initially, the activation energy derived from TGA-based Flynn–Wall–Ozawa analysis was input into the Arrhenius equation. This allowed us to design a predictable accelerated aging protocol at 150 °C that simulates equivalent service conditions at 70, 80, and 90 °C for periods of 1, 3, and 5 years. Following this, the crosslink density and elongation at break for each degradation stage were quantitatively determined through tensile, DMA, and TGA tests. These experimental data were then used to reverse-engineer the parameters for the Neo-Hookean, Mooney–Rivlin, and 2nd-order Yeoh hyperelastic models. The accuracy of each model was then evaluated by an L^2^-norm comparison.

Our findings verify that the Neo-Hookean model provides the best fit during the initial degradation phases, whereas the Mooney–Rivlin model excels in accuracy beyond the brittle transition point. Building on these findings, we derived a practical design guideline presented as a contour map, which allows for the selection of the most suitable material model based on two key criteria: crosslink density and strain at maximum tensile strength.

**Figure 1:**
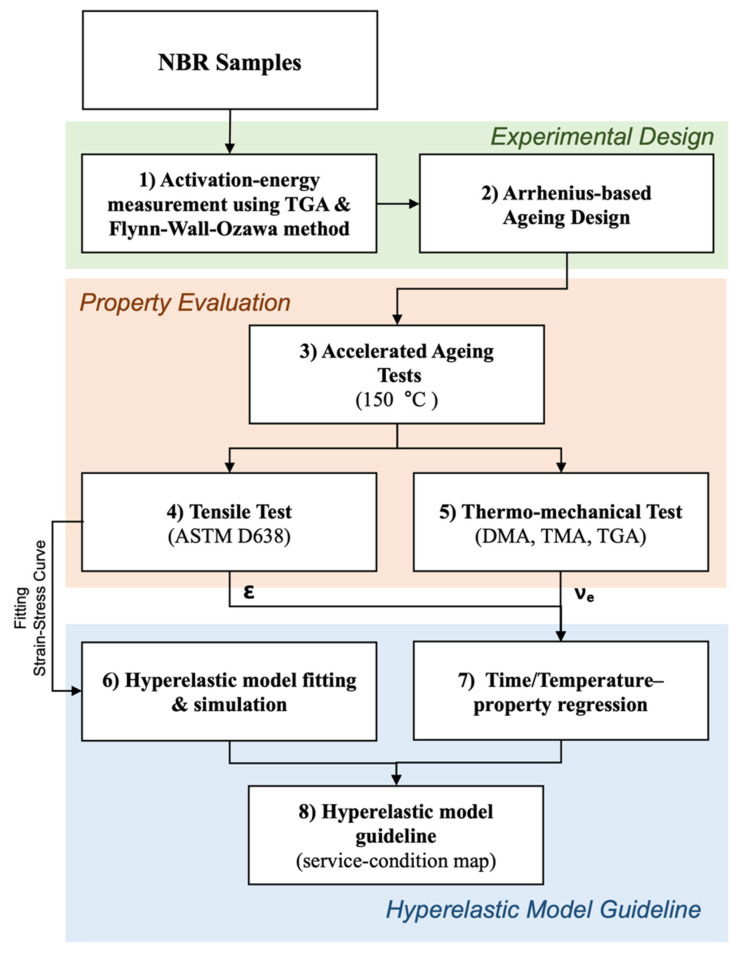
Workflow from accelerated-aging design to hyper-elastic model-selection guideline for NBR seals.

The overall workflow of this research is outlined in Figure 1. The structure of this paper is as follows: Section 2 reviews the Arrhenius-based theory for lifetime prediction; Section 3 presents the results from accelerated aging tests and material characterization; Section 4 details the suitability analysis of each model; Section 5 establishes the degradation boundaries and model transition criteria and discusses the broader engineering implications. Ultimately, this work intends to offer practical guidance for designing NBR seals for automotive and renewable energy applications that operate in high-temperature and high-pressure conditions.

## 2. Determination of Equivalent Service Life of NBR Using Activation Energy

The long-term evolution of mechanical properties and the lifetime of rubber materials can be determined via accelerated aging experiments founded on kinetic models, which leverage the laws governing time- and temperature-dependent property changes [27,28]. In this work, the Arrhenius equation was employed to determine the required duration for accelerated aging tests by establishing a conversion relationship between actual and accelerated service conditions.

As the long-term degradation of rubber follows a temperature-dependent reaction rate, it can be modeled using the Arrhenius equation [29,30]. The Arrhenius equation is given as follows in Equation (1).

(1) $$k=A^{\prime }{exp}^{\left(\frac{E_{a}}{RT}\right)}$$

where *k* represents the chemical reaction rate, $E_{a}$ is the activation energy (kJ mol^−1^), *R* is the universal gas constant, *T* denotes the absolute temperature (*K*), and *A* is the pre-exponential factor. A key prerequisite for calculating the accelerated ageing duration for a target service life with the Arrhenius equation is determining the material’s characteristic activation energy. To this end, we conducted thermogravimetric analysis (TGA) on NBR specimens and applied the isoconversional Flynn–Wall–Ozawa (FWO) method using multiple heating rates (5, 10, 20, and 50 °C/min) [31,32,33]. The FWO equation is given as follows in Equation (2).

(2) $$logF\left(\alpha \right)=\log\left(\frac{AE_{a}}{R}\right)-log\beta -2.315-0.4567\frac{E_{a}}{RT}$$

where $F\left(\alpha \right)$ is the integral form of the conversion function, A is the pre-exponential factor, $E_{a}$ is the activation energy (kJ mol^−1^), *R* is the universal gas constant (8.314 J mol^−1^ K^−1^), *β* is the constant heating rate (°C/min), and *T* is the absolute temperature (*K*). The numerical constants, −2.315 and −0.4567, are empirically derived coefficients from Doyle’s approximation of the temperature integral, which is a key step in the derivation of the FWO method [34].

The activation energy, $E_{a}$, was determined from the slope of the regression line on a plot of ln(*β*) against 1/*T*. The coefficients of determination (*R*^2^) for the regression lines at four distinct conversion rates (*α* = 5, 10, 20, and 50%) were all above 0.99, indicating a strong linear fit. As the conversion (*α*) progressed, the calculated *E_a_* increased from 60 to 232 kJ mol^−1^, yielding a mean value of 131 kJ mol^−1^. Detailed weight-loss curves and regression results for the Flynn–Wall–Ozawa (FWO) method are provided in Supporting Information Figure S1 and Table S1.

By substituting the calculated activation energy into the Arrhenius equation, we determined the necessary durations for the accelerated aging tests conducted at 150 °C, selected based on TGA evidence of no meaningful mass loss below ~300 °C and the validated upper limit of our DMA/TMA workflow (≤150 °C). The resulting accelerated aging times, which are designed to simulate 1, 3, and 5 years of service life at operating temperatures of 70 °C, 80 °C, and 90 °C, are detailed in Table 1. The specimen identification scheme used in this study, #1 through #10, corresponds to these incremental aging durations, beginning with the un-aged specimen (#1) and culminating with the specimen aged for a maximum of 91.8 h (#10). This nomenclature is maintained consistently in all following sections.

## 3. Accelerated Ageing Test and Evaluation of Material Property Changes in NBR with Degradation

### 3.1. Experimental Equipment and Test Conditions

All experimental equipment and key test conditions used in this study are summarized in Table 2. To evaluate the mechanical changes in the acceleratedly aged NBR specimens, tensile tests were first performed on five specimens for each condition in accordance with the ASTM D638 standard [35]. From the five resulting stress-strain curves, the specimen exhibiting the median behavior was selected as the representative specimen. Subsequently, thermogravimetric analysis (TGA), dynamic mechanical analysis (DMA), and thermomechanical analysis (TMA) were all performed sequentially on this same specimen. This in-depth analysis of a single representative specimen was intended to ensure data consistency while simultaneously enhancing both experimental efficiency and reliability.

To ensure the appropriateness of selecting the median specimen for subsequent TGA, DMA, and TMA tests, a statistical reproducibility analysis was conducted on the tensile test results. The coefficients of variation for ultimate tensile strength, elongation at break, and elastic modulus across all groups were below 2.6%, indicating low variability among specimens. Moreover, the deviation of the median value from the group mean was within 0.77%, confirming that the median specimen is statistically representative of the mechanical behavior of the entire group.

### 3.2. Results and Discussion

**Figure 2:**
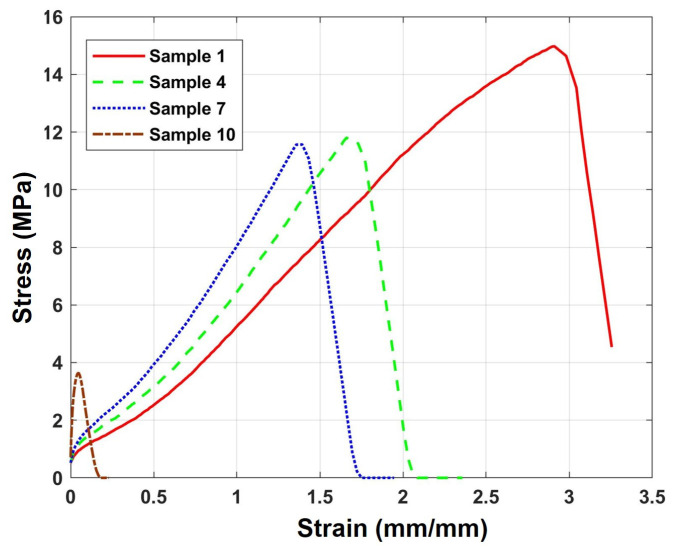
Representative stress-strain curves of NBR specimens at selected aging stages.

The mechanical properties of NBR were evaluated as a function of aging time. The stress-strain curves for Samples #1, #4, #7, and #10 are presented in Figure 2, while the ultimate tensile strength, elastic modulus, and elongation at break are summarized in Table 3. As the aging time increased, the elongation at break decreased sharply from 273% (a strain of 2.73) to 5.5% (a strain of 0.055), whereas the elastic modulus increased linearly from 6.01 MPa to 76.71 MPa. This behavior is a clear demonstration of typical embrittlement, where the material loses flexibility and becomes stiffer. Such changes are characteristic of the thermal-oxidative aging process in NBR, during which oxidative cross-linking reactions restrict the mobility of the polymer network [36,37]. On the other hand, the tensile strength showed no significant change in the initial stages of aging but exhibited a decreasing trend in the later stages (see Supporting Information Figure S2).

**Figure 3:**
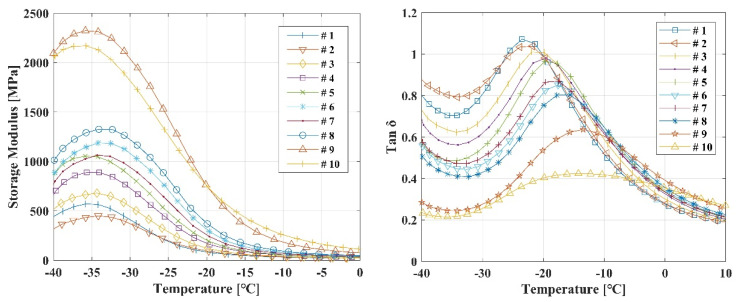
Temperature-Dependent Dynamic Mechanical Properties of NBR during Thermal Aging.

To elucidate the underlying cause of the observed changes in mechanical properties, DMA was carried out. Storage modulus and crosslink density values extracted from the rubbery plateau region (Tg + 50 °C) are summarized in Table 4. Detailed DMA measurement results are shown in Figure 3. With progressive aging, an increasing trend in the storage modulus and a decreasing trend in the tan δ peak were observed. This behavior indicates an increase in the crosslink density between the polymer chains. Indeed, the crosslink density, calculated based on Flory’s theory, was found to increase linearly with respect to aging time.

**Figure 4:**
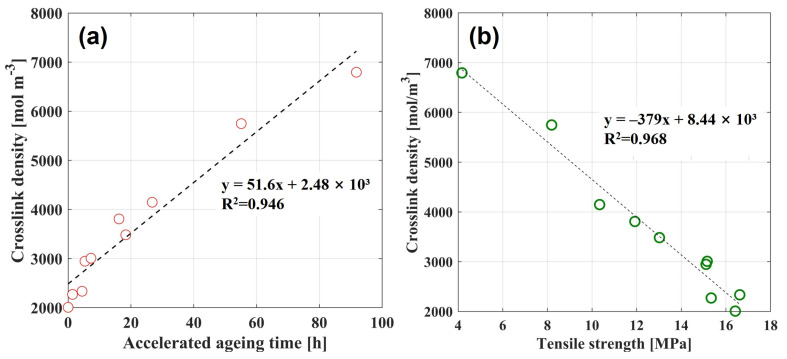
(**a**) Linear increase in crosslink density as a function of accelerated aging time. (**b**) Corresponding decrease in tensile strength as a function of crosslink density.

Figure 4 visually represents the significant relationship between the rise in crosslink density and the decline of mechanical properties. As depicted in Figure 4a, the crosslink density derived from DMA analysis shows a clear linear increase as a function of accelerated aging time. Figure 4b illustrates the correlation between crosslink density and tensile strength. A Pearson correlation analysis confirmed a statistically significant, strong negative correlation between tensile strength and crosslink density (r = −0.986, *p* < 0.001). Similarly, a very strong and statistically significant negative correlation was found between elongation at break and crosslink density (r = −0.965, *p* < 0.001). These statistical findings confirm that the deterioration in the material’s strength and flexibility is significantly associated with the increase in crosslink density caused by thermal degradation.

TGA and TMA were conducted to investigate further changes in thermal characteristics due to degradation. According to the TMA results, neither the glass transition temperature (Tg) nor the coefficient of thermal expansion (CTE) displayed a distinct trend as a function of aging time. Thermal expansion coefficients and Tg values for all aging conditions are listed in Supporting Information Table S2. Similarly, TGA revealed no significant shift in the primary decomposition temperature of the aged specimens; however, a tendency for reduced weight loss was observed during the initial dehydration phase (refer to Supporting Information Table S3). This implies that some chemical transformations had already taken place during the accelerated aging protocol.

## 4. Selection and Validation of Optimal Hyperelastic Models for Design Reliability

### 4.1. Hyperelastic Constitutive Models

Materials characterized by non-linear stress-strain behavior are known as hyperelastic materials. These materials, of which rubber is a typical example, can sustain large elastic deformations and fully recover their initial configuration upon the removal of the load. The mechanical response of such materials is described using hyperelastic models, where the stress-strain relationship is derived from a scalar function known as the strain energy density function. The strain energy density function, *W*, quantifies the energy stored per unit of undeformed volume, and the specific mathematical expression for *W* constitutes the hyperelastic model itself [38].

The strain energy density function can be generally approximated as follows in Equation (3) [39].

(3) $$W=W(I_{1},I_{2},I_{3})$$

where $I_{1},I_{2},I_{3}$ are the three invariants of the Green stress tensor;

(4) $$I_{1}=\lambda_{1}^{2}+\lambda_{2}^{2}+\lambda_{3}^{2}$$

(5) $$I_{2}=\lambda_{1}^{2}\lambda_{2}^{2}+\lambda_{2}^{2}\lambda_{3}^{2}+\lambda_{3}^{2}\lambda_{1}^{2}$$

(6) $$I_{3}=\lambda_{1}^{2}\lambda_{2}^{2}\lambda_{3}^{2}$$

Here, $\lambda_{1}^{2}\lambda_{2}^{2}\lambda_{3}^{2}$ is represented by the main elongation.

To characterize the hyperelastic constitutive response of the rubber, this research employed three models: the Neo-Hookean model, the 5-parameter Mooney–Rivlin model, and the second-order Yeoh model. A brief overview of these three models is provided below.

#### 4.1.1. Neo-Hookean Model

The Neo-Hookean model, which is founded on molecular theory, represents the simplest form of hyperelasticity. It is considered an extension of Hooke’s law to large deformations [40] and characterizes the material’s hyperelastic response with just one independent material constant. The mathematical expression for the Neo-Hookean model is given as Equation (7) [41].

(7) $$W=C_{1}(I_{1}-3)$$

where *C*_1_ is a material constant, and *I*_1_ is the first invariant of the left Cauchy-Green deformation tensor.

#### 4.1.2. Yeoh Secondary Model

The Yeoh model is a phenomenological model similar to the Mooney–Rivlin model, but with the defining characteristic that its strain energy density function depends exclusively on the first strain invariant, $I_{1}$ [42]. The mathematical form of the second-order Yeoh model is presented as Equation (8) [43,44].

(8) $$W=C_{10}\left(I_{1}-3\right)+C_{20}{\left(I-3\right)}^{2}+\frac{1}{d_{10}}{\left(J-1\right)}^{2}+\frac{1}{d_{20}}{\left(J-1\right)}^{4}$$

where $I_{1}$ is the first principal strain invariant (sum of the principal strains), *J* is the strain Jacobian (a measure of the volume change produced by deformation), *C*_10_ and *C*_20_ are material constants (units of stress), and $d_{10}$ and $d_{20}$ are compressibility parameters (units of inverse stress).

#### 4.1.3. Mooney–Rivlin 5-Parameter Model

The Mooney–Rivlin model is considered one of the foundational hyperelastic models, alongside the Yeoh model. Owing to its relative simplicity and effectiveness, it has become one of the most popular models for analyzing nonlinear elastic behavior. The model’s versatility allows it to be extended to capture more complex deformation modes by increasing the number of parameters [45]. The specific form of the 5-parameter Mooney–Rivlin model employed in this research is given by Equation (9). The material constants *C*_10_, *C*_01_, *C*_11_, *C*_20_, and *C*_02_ are determined from the experimental data.

(9) $$\begin{array}{cc} W=2C_{10}(\lambda -\frac{1}{\lambda }) & +2C_{01}\left(\lambda -\frac{1}{\lambda^{3}}\right)+6C_{11}\left(\lambda^{2}-\lambda -1+\frac{1}{\lambda^{2}}+\frac{1}{\lambda^{3}}-\frac{1}{\lambda^{4}}\right)\\ & +4C_{20}\lambda \left(1-\frac{1}{\lambda^{3}}\right)\left(\lambda^{2}+\frac{2}{\lambda }-3\right)+4C_{02}(2\lambda +\frac{1}{\lambda^{2}}-3)(1-\frac{1}{\lambda^{3}}) \end{array}$$

The fitted hyperelastic constants for the Neo-Hookean, Yeoh, and Mooney–Rivlin models were obtained from tensile test data. These values are summarized in Table 5. They were then used as input parameters for subsequent FEA simulations to evaluate model accuracy.

### 4.2. FEA Model and Accuracy Validation Method

**Figure 5:**
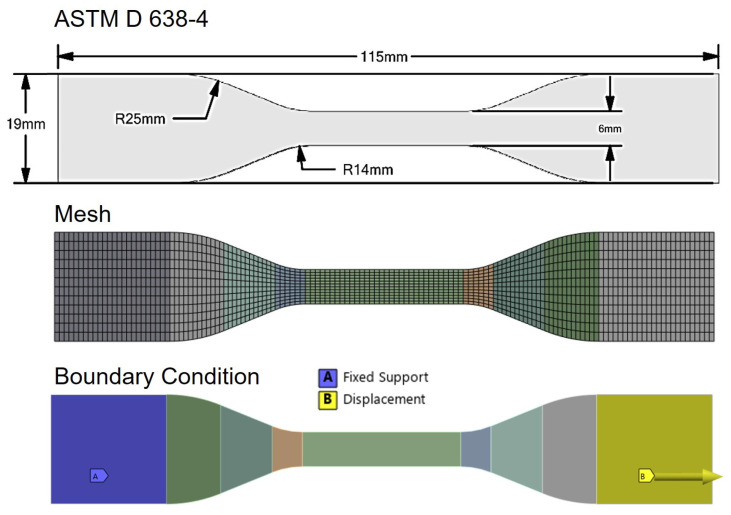
Finite element analysis (FEA) model for the validation of hyperelastic models.

To assess the accuracy of the selected hyperelastic models, we constructed a finite element (FE) simulation model for mechanical property tests. Figure 5 illustrates the FE model of the test specimen, including the generated mesh and the applied boundary conditions. The model’s geometry was designed in accordance with the ASTM D 638 Type IV standard, making it identical to the physical specimens used in the experimental tensile tests. To ensure precise calculations of deformation and stress, a more refined mesh was utilized in the central gauge section. The experimental conditions were simulated by constraining one end of the specimen with a fixed support and applying a prescribed displacement to the opposite end. The numerical analysis was conducted using ANSYS Academic Research software (ANSYS Workbench 2024 R2), made available through the Convergence Parts and Materials Core Research Support Center.

**Figure 6:**
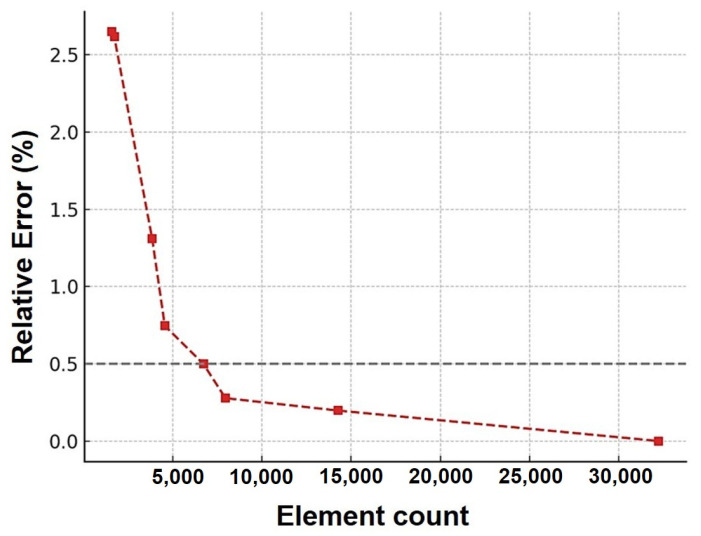
Mesh convergence analysis showing relative error in maximum von Mises stress versus element count. The red dashed line indicates the relative error trend, and the gray dashed line represents the 0.5% error threshold.

A mesh sensitivity evaluation was undertaken before validating the finite element models. Maximum von Mises stress in the gauge section was calculated for element counts between 1584 and 32,224. The 32,224-element mesh was used as the reference. The relative error fell to 0.5 percent at around 6720 elements, which was taken as the point of convergence. A mesh of 6720 elements was used for all further simulations to provide a practical balance between accuracy and computational efficiency. Figure 6 shows the convergence behavior with relative error in maximum von Mises stress versus element count and the 0.5 percent threshold for optimal mesh density.

To quantitatively evaluate which hyperelastic model best fits the experimental data, the L2-Norm was employed as a measure of error. In linear algebra, a norm is defined as a function that assigns a strictly positive length or magnitude to each vector in a vector space. The L2-Norm, often associated with the principle of Least Squares Error, works by minimizing the sum of the squared differences between the target (experimental) and estimated (model-predicted) values [46].

The mathematical expression for the L2-Norm is given in Equation (10).

(10) $$S=\sqrt{\sum_{n=1}^{i}{\left(y_{1}-f\left(x_{1}\right)\right)}^{2}}$$

where $y_{1}$ represents the target (experimental) value and $x_{1}$ denotes the estimated (model-predicted) value. For each sample in this study, ten discrete data points were selected from its experimental stress-strain curve. The L2-Norm was then used to evaluate which of the Mooney–Rivlin, Yeoh, and Neo-Hookean models more closely approximated the actual experimental results.

### 4.3. Model Accuracy Assessment and Optimal Model Selection

**Figure 7:**
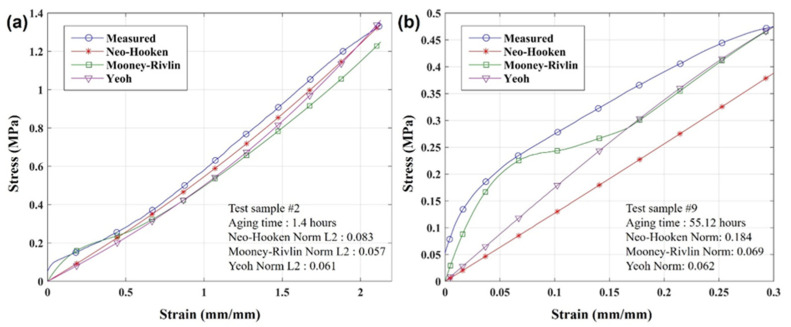
Validation of hyperelastic models against experimental stress-strain curves at different degradation stages: (**a**) early-stage ageing (1.4 h) and (**b**) late-stage ageing (55.12 h).

As representative examples, Figure 7 illustrates a comparison between the experimental stress-strain curves for two specimens, Sample #2 (less aged) and Sample #9 (more aged), and the corresponding curves predicted by the hyperelastic models through finite element analysis. The degree of approximation for each model was quantified by calculating the L2-Norm at the points indicated by markers. A comprehensive summary of the L2-Norm values for all specimens across the different aging durations is provided in Table 6. A lower L2-Norm value signifies a better agreement between the model prediction and the experimental results.

The goodness-of-fit analysis for the hyperelastic models across various degradation conditions demonstrated that during the initial phase of degradation (up to approximately 7 h of accelerated aging), the Neo-Hookean model yielded the best agreement with experimental data. This suggests that a simpler constitutive model is sufficient for effectively predicting the mechanical response in the early stages. Conversely, for advanced stages of degradation (16.2 h and beyond), the 5-parameter Mooney–Rivlin model exhibited superior accuracy, indicating the necessity of a more sophisticated model incorporating higher-order terms to capture the material’s behavior.

These findings underscore the importance of a strategic approach to selecting a suitable hyperelastic model according to the material’s degradation state. Specifically, an efficient methodology would involve applying the Neo-Hookean model for materials in the early stages of degradation and transitioning to the Mooney–Rivlin model as the material becomes more severely degraded.

## 5. Proposal for a Comprehensive Hyperelastic Model Selection Guideline

### 5.1. Defining the Degradation Inflection Point and Embrittlement Boundary

Based on the experimental results from Section 4, this section synthesizes the analysis of mechanical and thermal property changes, derived from Arrhenius-based NBR accelerated aging tests, with the accuracy evaluation results of hyperelastic models from Finite Element Analysis (FEA). Based on this, an integrated hyperelastic model selection guideline is proposed to improve the reliability of NBR components in real-world engineering design.

A key finding of this research is the observation of a critical inflection point at 16.2 h of accelerated aging (at 150 °C), which corresponds to 3 years of service life at 80 °C. At this juncture, the material’s embrittlement significantly intensifies. This was evidenced by a sharp drop in the elongation at break to 124.6% (a strain of 1.246) from 181.3% (a strain of 1.813) recorded at the prior stage (7.27 h). The underlying cause for this shift in behavior is a critical increase in crosslink density. Upon reaching 16.2 h, the crosslink density surged to over 3800 mol m^−3^ from 3007 mol m^−3^ at the previous stage, thereby restricting polymer chain mobility and stripping the material of its flexibility. This qualitative transformation in the material directly influenced the selection of the FEA model; it was confirmed that at this turning point, the optimal constitutive model transitions from the simpler Neo-Hookean to the more complex 5-parameter Mooney–Rivlin model.

### 5.2. Guideline Based on Service Conditions: The Degradation Map

To establish a clear guideline for selecting hyperelastic models, we conducted a linear regression analysis based on our findings, targeting three key parameters: strain at maximum tensile strength, ultimate tensile strength, and crosslink density. The objective was to quantify a predictive model for these material properties as a function of service conditions (temperature and time). The outcomes of the linear regression analysis for these three variables are summarized in Table 7.

Upon comparing the three properties, strain at maximum tensile strength exhibited the strongest regression fit and the highest predictive capability. Crosslink density, in turn, proved to be a valuable auxiliary indicator for interpreting behavioral changes arising from structural transformations in the material. While ultimate tensile strength is a conventional metric for performance assessment, its predictive power in this context was found to be relatively weak. Consequently, this study established model selection criteria primarily based on strain at maximum tensile strength and crosslink density.

In this research, we define the embrittlement boundary for NBR by the conditions where the strain at maximum tensile strength is ≤1.5 (mm/mm) or the crosslink density is ≥3300 mol m^−3^. This threshold marks the onset of brittle behavior in the material. Critically, it also corresponds to the domain where the more complex Mooney–Rivlin model begins to provide a superior fit to the experimental data.

**Figure 8:**
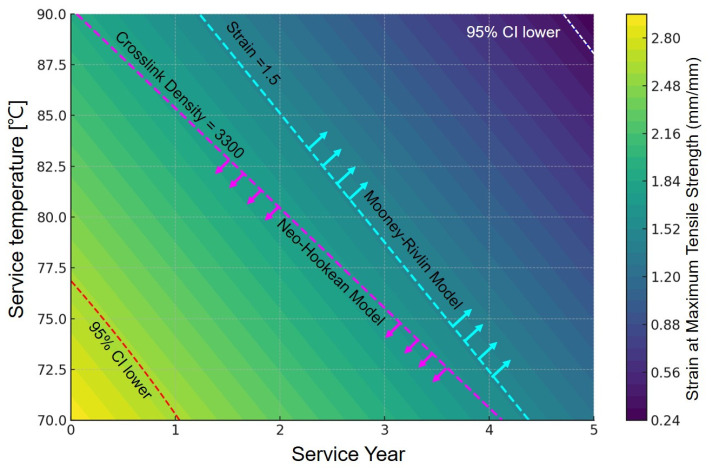
Design-oriented degradation map and model selection guideline for NBR based on service conditions (temperature and time). The arrows indicate the transition boundary and direction of the applicable hyperelastic model (from Neo-Hookean to Mooney–Rivlin).

This concept is visually synthesized in Figure 8, which presents a contour map of strain at maximum tensile strength against service conditions (years and temperature). The boundary line for Strain = 1.5 (mm/mm), indicated by a dashed line, delineates the zone of rapid ductility loss. The superposition of this boundary with the high crosslink density region identifies a critical zone of concurrent structural and mechanical degradation. This suggests that within this zone, material embrittlement must be assumed, and a transition in the constitutive model is warranted.

However, note that the embrittlement boundary delineated in this map (i.e., Crosslink Density ≥ 3300 mol m^−3^) was determined for the specific NBR compound tested in this study. This value can vary depending on the rubber’s formulation, such as the type and amount of additives. Therefore, applying this map directly to other NBR systems would require preliminary validation.

Therefore, the hyperelastic model selection guideline framework proposed herein can serve as a foundational basis for developing future intelligent model selection systems that account for material degradation. Such advancements are expected to enhance the accuracy of nonlinear analyses for rubber components, thereby contributing to more robust and reliable design optimization.

### 5.3. Discussion

This study successfully establishes an integrated workflow to quantitatively analyze the state of degradation in NBR and, based on this analysis, select the optimal hyperelastic model. The central finding is that as degradation progresses, the most accurate predictive model transitions from the simpler Neo-Hookean model to the more complex Mooney–Rivlin model. This demonstrates that a data-driven methodology can fill the technical gap identified in the introduction, where model selection has traditionally relied on empirical engineering judgment.

This model transition can be rationalized by the physical evolution of the material. In its early degradation stages, NBR possesses a relatively uniform polymer network, the behavior of which can be sufficiently captured by the statistical mechanics-based Neo-Hookean model [47]. However, with continued aging, additional cross-linking reactions lead to a linear increase in crosslink density. This increased density constrains polymer chain mobility and enhances the network’s non-ideality, making its behavior too complex to be described by a single-parameter ($I_{1}$) model. As a result, the Mooney–Rivlin model [48], which incorporates the second strain invariant ($I_{2}$) into its strain energy function, demonstrates superior predictive accuracy in these later stages.

The ‘degradation map’ (Figure 8), which is the culmination of this analysis, provides designers with a tangible and practical guideline. For instance, an engineer can input a component’s target service life and temperature into the map to instantly assess whether it falls within the embrittlement zone. This allows for the informed adoption of a more sophisticated model, such as the Mooney–Rivlin model, for Finite Element Analysis (FEA), thereby significantly enhancing design reliability.

Although this study puts forth a successful framework for model selection for NBR, it is crucial to recognize its limitations to generalize the findings and broaden their applicability. The main limitations are that accelerated aging was conducted at a single temperature (150 °C), which may not fully represent the influence of diverse thermal conditions, and that the study did not account for chemo-mechanical interactions with substances like oil, which are prevalent in actual service environments. To address these limitations, future research should focus on validating the Arrhenius model’s predictive power across multiple temperature points. Moreover, the framework needs to be expanded by conducting combined-environment tests (e.g., simultaneous thermal and oil exposure) to build more robust degradation models and design guidelines that more faithfully represent real-world powertrain conditions.

## 6. Conclusions

This study sought to analyze the degradation behavior of industrial Nitrile-butadiene rubber (NBR) and to propose an integrated workflow for high-fidelity Finite Element Analysis. To this end, a TGA-derived activation energy (*E_a_* = 131.34 kJ mol^−1^) was applied to the Arrhenius model to systematically design accelerated aging tests. Subsequent characterization via DMA and tensile tests quantitatively confirmed that as degradation proceeds, crosslink density increases linearly (from 2.0 × 103 to 6.8 × 103 mol m^−3^). This was identified as the primary cause of embrittlement, evidenced by a drastic reduction in elongation at break from 273% (a strain of 2.73) to 4% (a strain of 0.04). Moreover, an L2-Norm error analysis demonstrated that the Neo-Hookean model provides the most accurate predictions for early-stage degradation, whereas the Mooney–Rivlin model excels for advanced degradation states. By integrating these findings, this research culminates in a practical design guideline, presented as a degradation map, that enables the selection of an optimal hyperelastic model based on real-world service conditions (temperature and time).

However, despite the validity of the proposed framework, this study has several limitations that suggest directions for future research. First, the accelerated aging was conducted at a single temperature (150 °C), meaning the Arrhenius model’s predictive accuracy was not fully validated across a wider range of thermal conditions. Second, this study focused solely on thermo-oxidative aging, whereas real-world service environments for NBR often involve synergistic effects from contact with substances like oil or from sustained mechanical stress. Furthermore, the brittle boundary value presented in this study (crosslink density ≥ 3300 mol m^−3^) is based on the experimental results of the specimens used, and it may vary depending on the actual rubber formulation, so further research on more diverse NBR specimens is needed. Finally, as this study’s characterization was centered on mechanical properties, further investigation into the chemical and morphological changes on the material’s surface, for instance, through techniques like FTIR or SEM, is warranted for future research.

The predictive accuracy of FEA for rubber components is critically dependent on the suitability of the chosen material model and its input parameters. In light of this, the integrated approach proposed herein facilitates a paradigm shift from conventional, experience-based design to a data-driven, predictive methodology. This work is therefore expected to make a substantial contribution to enhancing the accuracy of long-term reliability assessments and the overall design of components based on rubber materials.

## Figures and Tables

**Table 1:** Accelerated-aging durations corresponding to the target service times and temperatures.

Test #	1	2	3	4	5	6	7	8	9	10
Target service time [year]	Unaged	1	3	1	5	3	1	5	3	5
Target service temperature [°C]	Unaged	70	70	80	70	80	90	80	90	90
Accelerated-ageing time [h]	Unaged	1.4	4.45	5.35	7.27	16.2	18.35	26.77	55.12	91.77

**Table 2:** Experimental equipment and test conditions.

Category	Equipment/Model	Manufacturer (Country)	Main Test Conditions	Standard/Mode and Application
Accelerated Ageing	Temperature Chamber SH-662	ESPEC (Osaka, Japan)	150 °C, constant high-temperature exposure	Ageing steps from 1.4 to 91.8 h based on Arrhenius equivalent lifetime design
Tensile Test	Universal Testing Machine KDMT-156	KDMT (Seoul, Republic of Korea)	ASTM D638 type IV specimens cross-head 300 mm min^−1^, measured until fracture ≥5 specimens per condition	-
TGA	TGA N-1000	Sinco (Seoul, Republic of Korea)	Sample 10–11 mg, heating rate 20 °C min^−1^ N_2_ atmosphere	Data acquisition for FWO activation energy calculation
DMA	Q800 DMA	TA Instruments (New Castle, DE, USA)	Tension mode, −40–150 °C heating rate 5 °C min^−1^ sinusoidal loading	Calculation of Storage/Loss modulus, tan δ, and cross-link density
TMA	Q400 TMA	TA Instruments (New Castle, DE, USA)	Expansion mode, −50–150 °C heating rate 5 °C min^−1^ 50 mN load	Measurement of Tg and CTE, rubbery plateau analysis

**Table 3:** Key mechanical properties of NBR specimens at different accelerated aging times.

Sample #	Ageing Time (h)	*σ* Max (MPa)	*ϵ* Break (%)	Elastic Modulus (MPa)
1	unaged	16.4 ± 1.16	273.46 ± 20.08	6.01 ± 0.14
4	7.2	15.11 ± 0.90	187.60 ± 12.89	8.06 ± 0.32
7	26.8	13.02 ± 0.32	135.51 ± 3.90	9.62 ± 0.30
10	91.8	4.17 ± 0.17	5.55 ± 0.66	76.71 ± 5.98

**Table 4:** Storage Modulus and Crosslink Density of Aged NBR Specimens from DMA at Tg + 50 °C.

Test #	Using Year	Using Temperature	Accelerated Aging Time	Storage Modulus at Rubbery Plateau Region (Tg + 50 °C)	Crosslink Density
	[year]	[°C]	[h·min]	[MPa]	[mol/m^3^]
1	0	0	0	14.53	2006.46
2	1	70	1.4	16.29	2269.97
3	3	70	4.45	16.70	2334.16
4	1	80	5.35	21.09	2946.25
5	5	70	7.27	21.45	3007.15
6	3	80	16.2	27.26	3807.24
7	1	90	18.35	24.99	3483.47
8	5	80	26.77	29.78	4146.81
9	3	90	55.12	41.39	5747.51
10	5	90	91.77	49.16	6794.17

**Table 5:** Hyperelastic constants of aged NBR for Neo-Hookean, Yeoh 2nd-order, and Mooney–Rivlin models at different accelerated aging times.

Hyperelastic Constant of NBR Neo-Hookean and Yeoh 2nd Order Model at Different Aging Time					
Test #	Neo-Hookean Model	Yeoh Secondary Order			
	Initial Shear Modulus	*C*_10_		*C*_20_	
	[Mpa]	[Mpa]		[Mpa]	
1	0.319	0.135		0.004	
2	0.339	0.142		0.006	
3	0.358	0.149		0.008	
4	0.365	0.158		0.007	
5	0.380	0.159		0.008	
6	0.402	0.190		0.005	
7	0.434	0.199		0.007	
8	0.473	0.243		−0.004	
9	0.872	0.607		−0.735	
10	3.967	3.266		−145.391	
**Hyperelastic Constant of NBR Mooney–Rivlin 5 Parameter at Different Aging Time**					
**Test #**	***C*** **_10_**	***C*** **_01_**	***C*** **_20_**	***C*** **_11_**	***C*** **_02_**
	**[Mpa]**	**[Mpa]**	**[Mpa]**	**[Mpa]**	**[Mpa]**
1	−1.65	2.04	0.04	−0.26	0.96
2	−2.28	2.74	0.14	−0.72	1.71
3	−2.59	3.08	0.23	−1.09	2.22
4	−3.23	3.80	0.38	−1.69	3.05
5	−2.76	3.30	0.21	−1.02	2.20
6	−4.87	5.60	1.17	−4.69	6.49
7	−4.63	5.35	0.96	−3.94	5.76
8	−7.50	8.41	3.86	−13.84	15.43
9	−44.41	46.60	467.01	−1213.50	818.52
10	−1140.14	1151.15	1,978,220.32	−4,174,395.94	2,205,309.53

**Table 6:** Comparison of Predictive Accuracy (L2-Norm Error) of Hyperelastic Models for Each Degradation Stage.

Test #	Neo-Hookean Model	Mooney–Rivlin 5 Parameter	Yeoh Secondary Order
1	0.0188	0.0129	0.0130
2	0.0019	0.0096	0.0053
3	0.0056	0.0165	0.0151
4	0.0046	0.0092	0.0102
5	0.0062	0.0158	0.0062
6	0.0069	0.0028	0.0095
7	0.0087	0.0068	0.0144
8	0.0138	0.0037	0.0126
9	0.0161	0.0017	0.0067
10	0.0015	0.0024	0.0064

**Table 7:** Regression analysis results for predicting mechanical properties of NBR as a function of service time (*Y*) and temperature (*T*).

Response Variable	Regression Equation	*R* ^2^	Adj. *R*^2^	Pred. *R*^2^	*p*-Value (Year)	*p*-Value (Temp)
Strain at Maximum Tensile Strength	6.57 − 0.3292*Y* − 0.0519*T*	80.2%	75.8%	59.6%	0.001	0.008
Ultimate Tensile Strength	37.85 − 1.266*Y* − 0.2718*T*	75.9%	70.5%	46.8%	0.003	0.006
Crosslink Density	−6196 + 517*Y* + 105.2	78.4%	73.6%	54.2%	0.002	0.005

## Data Availability

The original contributions presented in this study are included in the article. Further inquiries can be directed to the corresponding author.

## Supplementary Materials

The following supporting information can be downloaded at: https://www.mdpi.com/article/10.3390/polym17172316/s1, Figure S1: TGA Weight Loss and Flynn–Wall–Ozawa (FWO) Linear Regression for Activation Energy Determination; Figure S2: Evolution of Mechanical Properties of NBR with Accelerated Aging Time; Table S1: Activation Energy of NBR at Different Conversion Levels Based on FWO Method; Table S2: Thermal Expansion Coefficients and Glass Transition Temperatures of Aged NBR Specimens (TMA Results); Table S3: Thermogravimetric Analysis of Aged NBR Specimens: DTG Peak, Ignition, and Burnout Characteristics.

## Abbreviations

The following abbreviations are used in this manuscript:

NBR	Nitrile-butadiene rubber
FEA	Finite Element Analysis
TGA	Thermogravimetric analysis
DMA	Dynamic mechanical analysis
TMA	Thermomechanical analysis
FWO	Flynn–Wall–Ozawa
CTE	Coefficient of thermal expansion
Tg	Glass transition temperature
*W*	Strain energy density function
*E_a_*	Activation energy
*I*_1_, *I*_2_, *I*_3_	Invariants of the Green stress tensor
*k*	Chemical reaction rate
*R*	Universal gas constant
*R*^2^	Coefficient of determination
*T*	Absolute temperature
*α*	Conversion rate
*β*	Constant heating rate
*ϵ**_break_*	Strain at break/Elongation at break
*λ*	Main elongation
*ρ_x_*	Crosslink density
*σ_max_*	Ultimate tensile strength
