# Supplementary material for: Design-Oriented Degradation Mapping and Hyperelastic Model-Switch Guidelines for Nitrile-Butadiene Rubber Seals
Source: Polymers (Basel). 2025 Aug 27;17(17):2316. doi: 10.3390/polym17172316 (PMC12431286; doi:10.3390/polym17172316)
Supplement: Supplementary file 1 [file polymers-17-02316-s001.zip › polymers-3758634-supplementary.pdf]

Supplementary Materials

Design-oriented degradation mapping and hyperelastic model-switch guidelines for nitrile-butadiene rubber seals

Figure S1. TGA Weight Loss and Flynn–Wall–Ozawa (FWO) Linear Regression for Activation Energy Determination

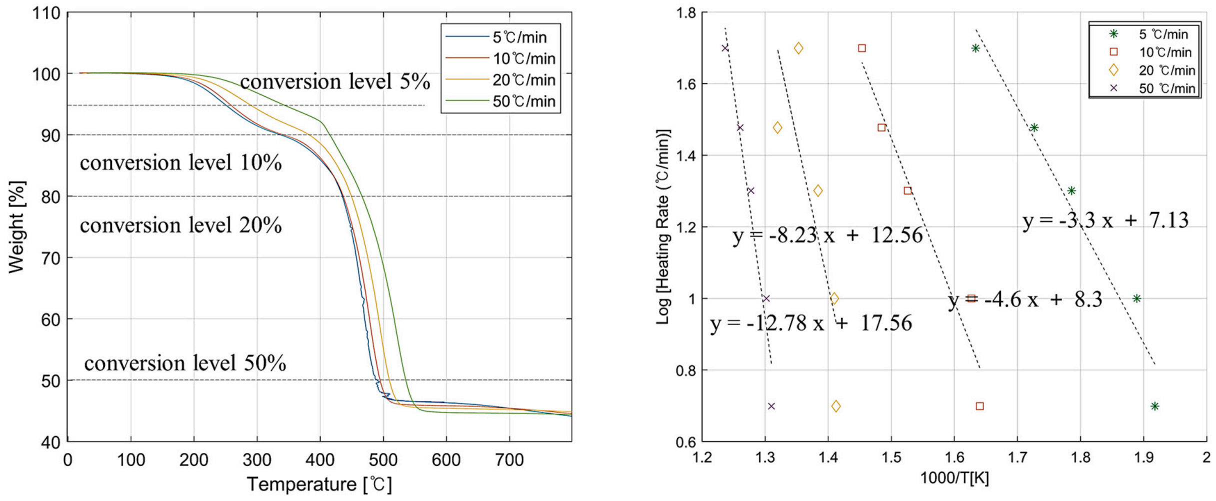

Table S1. Activation Energy of NBR at Different Conversion Levels Based on FWO Method

conversion level	Ea [kJ/mol]
**5**	59.95
**10**	83.15
**20**	149.8
**50**	232.46
**Average**	131.34

Figure S2. Evolution of Mechanical Properties of NBR with Accelerated Aging Time

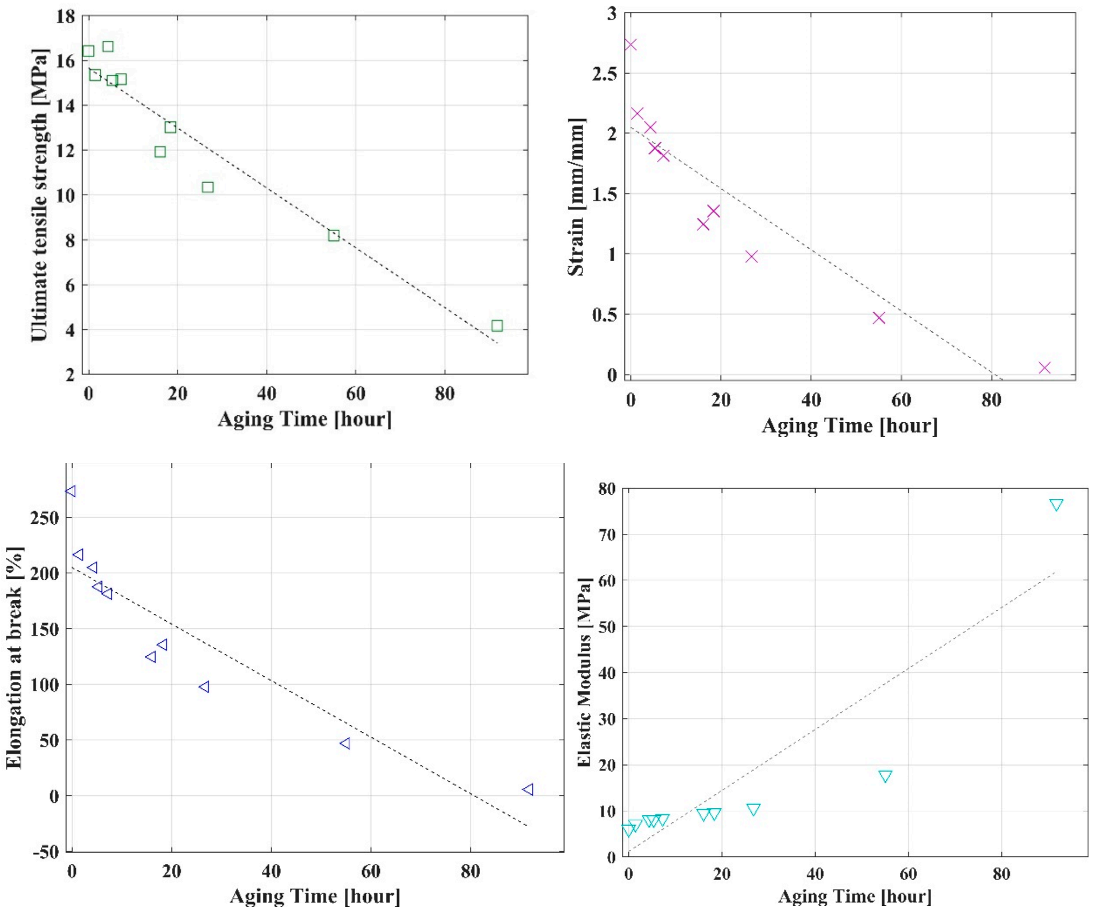

Table S2. Thermal Expansion Coefficients and Glass Transition Temperatures of Aged NBR Specimens (TMA Results)

Test #	using year	using temperature	acceleated Aging Time	thermal expansion coefficients	Glass transition temperature
	[year]	[℃]	[hour.min]	[ppm/℃]	[℃]
**1**	0	0	0	196.24	-22.83
**2**	1	70	1.4	215.38	-25.48
**3**	3	70	4.45	194.43	-26.25
**4**	1	80	5.35	189.721	-26.16
**5**	5	70	7.27	197.38	-27
**6**	3	80	16.2	207.34	-25.98
**7**	1	90	18.35	196.80	-25.51
**8**	5	80	26.77	199.02	-25.39
**9**	3	90	55.12	174.22	-24.6
**10**	5	90	91.77	209.696717	-22.9

Table S3. Thermogravimetric Analysis of Aged NBR Specimens: DTG Peak, Ignition, and Burnout Characteristics

Test #	using year [year]	using temperature [℃]	acceleated Aging Time [hour.min]	maximum peak temperature of DTG [℃]	Weight loss @maximum peak temperature of DTG [%]	Ignition Temperature [℃]	Weight loss @Ignition Temperature [%]	Burnout Temperature [℃]	Weight loss @ Burnout Temperature [%]
**1**	0	0	0	491.77	60.47	413.43	87.05	526.62	46.13
**2**	1	70	1.4	496.95	62.77	418.15	87.97	534.16	47.97
**3**	3	70	4.45	494.35	64.79	423.58	90.32	531.37	49.71
**4**	1	80	5.35	496.99	64.34	422.4	89.94	535.07	49.3
**5**	5	70	7.27	495.87	64.7	424.65	89.94	533.25	49.41
**6**	3	80	16.2	497.5	66.53	429.38	91.62	535.26	51.11
**7**	1	90	18.35	494.5	65.56	424.97	90.8	532.45	50.32
**8**	5	80	26.77	497.36	66.39	426.44	90.92	535.48	51.41
**9**	3	90	55.12	494.24	68.06	428.96	91.94	534.21	52.03
**10**	5	90	91.77	492.1	68.51	423.79	91.37	530.81	53.94
